# Surgical outcome following primary closure of auricular lacerations

**DOI:** 10.1007/s00405-025-09453-2

**Published:** 2025-05-15

**Authors:** Max Zwemstra, Simon Geerse, Maarten de Wolf, Fenna Ebbens, Cas Smits, Erik van Spronsen

**Affiliations:** Department of Otorhinolaryngology, Otolaryngology-Head and Neck Surgery, Amsterdam University Medical Centers, Location Academic Medical Center, Meibergdreef 9, 1105 AZ Amsterdam, The Netherlands

**Keywords:** Ear laceration, Primary closure, Success rate

## Abstract

**Purpose:**

To evaluate the aesthetic outcomes of primary closure of auricle lacerations in the emergency department.

**Methods:**

In this prospective case study in our tertiary referral center we included all patients with an auricular laceration without complete avulsion. Wound treatment involved thorough cleaning and primary closure. Photos of the auricle were taken at various stages for evaluation. To evaluate the achieved results after 3 months, a success classification system with four classes was developed: (1) full success, no irregularities, (2) partial success, only minor irregularities, (3a) unsuccessful, major irregularities or (3b) unsuccessful, (partial) necrosis.

**Results:**

Most patients demonstrated successful outcomes (60.7% full success and 32.1% partial success), with helical involvement being associated with inferior results. The interobserver percent agreement of the classification system was 88%. The Fleiss’ kappa coefficient was 0.570, *p* < 0.001.

**Conclusion:**

Primary closure of ear lacerations results in a high success rate. We have introduced a revised classification system to qualify the success of treatment of the auricle laceration. Involvement of the helix is correlated with inferior surgical results three months after surgical closure.

Trial registration number and date of registration: reference number W21_387 # 21.432, September 9, 2021.

## Introduction

The auricle, or pinna, is composed of thin dermis adhered to a unique cartilaginous framework with minimal subcutaneous adipose tissue [1]. The average height and width of a fully formed auricle are respectively 6 and 4 cm. The auricle is composed of a complex framework of convex and concave surfaces [1]. The angle of protrusion of the auricle from the skull base is approximately 25 to 30 degrees. The auricle collects, amplifies and transfers soundwaves from the external environment to the ear canal [2]. The unique shape and folds of the auricle alter incoming sound waves, generating spectral cues that assist the brain in localizing sound sources, particularly in distinguishing between anterior–posterior and vertical directions [3]. Blood supply to the auricle is provided by the superficial temporal artery (STA) and the posterior auricular artery (PTA) [4, 5].

Due to its prominent, lateral position on the human head, injuries to the auricle are relatively common in facial trauma [6]. Most cases of injury to the auricle occur between the age of 11–40 years. Men outnumber women at a ratio of 2 to 1 [7]. The most frequent causes for auricular trauma are traffic accidents, accidents at home, sports injuries and assaults, including human and dog bites [6, 8]. Understanding of the auricular anatomy and development are crucial for successful reconstruction [6].

Besides functional anatomy, the auricle is critical in facial aesthetics. Acquired auricular deformities can have a significant impact on psychosocial functioning and self-esteem [9].

Auricular lacerations can lead to permanent cosmetic deformities [1, 6].

Auricle lacerations can be subdivided in ear lacerations with or without involvement of cartilage and in partial or total avulsion [6]. There is a prevailing consensus among otolaryngologists globally that primary closure yields favorable outcomes for healing lacerations of the auricle. This is based on studies published in the early sixties and eighties [10, 11]. In this retrospective study we intend to reevaluate the results of primary closure in the emergency department setting.

Our objective is to retrospectively review the esthetic results of primary closure of ear lacerations in the emergency department setting using a success classification system.

## Participants and methods

### Subjects

We included a total of 39 patients presented with a laceration of the auricle in the emergency department or the otolaryngology outpatient clinic of our tertiary referral hospital between January 2017 and November 2023. Risk factors for impaired wound healing were collected (Table 1). All participants agreed to participate in the study. The study protocol was in accordance with the Helsinki declaration and was approved by the ethical review board of the hospital.

**Table 1:** Baseline characteristics. *Classification based on the classification of Lavasani [1]

	Total	Total included for analysis
	*n* = 39	*n* = 28
Mean age (years)	45 (4–98)	40 (4–98)
Sex		
* Female*	12 (31%)	8 (29%)
* Male*	27 (69%)	20 (71%)
Side of laceration		
* Left*	21 (54%)	13 (46%)
* Right*	18 (46%)	15 (54%)
Risk factors		
* Current cigarette smoking*	6 (15%)	4 (14%)
* Cardiovascular disease*	11 (28%)	6 (21%)
* Diabetes*	1 (3%)	1 (4%)
Cause of ear trauma		
* Injury at home*	27 (69%)	20 (71%)
* Traffic accident*	5 (13%)	4 (14%)
* Fight*	4 (10%)	1 (4%)
* Bite*	2 (5%)	2 (7%)
* Sports accident*	1 (3%)	1 (4%)
Auricular injury classification *		
Cartilage sparing injury	12 (31%)	6 (21%)
Partial avulsion with wide pedicle	24 (62%)	20 (71%)
Partial avulsion with narrow pedicle	3 (8%)	2 (7%)
Complete avulsion	0	0
Helix involvement	24 (62%)	18 (64%)

### Wound treatment

On initial survey, auricular injuries were often covered by blood and debris. Thorough antiseptic cleaning of the wound, including removal of gross contaminants was preceded by primary closure of the laceration [6]. Local anesthesia was administered using articaine hydrochloride 40 mg, adrenaline (as hydrochloride) 5 microg. Visible necrotic tissue was debrided if necessary. If the auricle laceration was comprised to the skin, primary closure with non-resolvable sutures (Ethilon® 5–0 suture) was obtained. If skin and cartilage were involved in the auricle laceration, the laceration was closed in two layers. We used resolvable sutures (Vicryl ® 4–0 suture) for the cartilage and non-resolvable sutures (Ethilon® 5–0 suture) to suture the skin. In cases where primary skin closure was not possible, it was the physicians experience to choose the best method of closure (e.g., wig excision of a small piece of cartilage) keeping the unique shape of the auricle in mind. All patients were administrated for an amoxicillin-clavulanic acid course of 7 days.

### Data collection

Of all included patients, photos of the auricle were obtained by the physician after antiseptic cleaning (primary survey), after wound closure, after 1 week and after three months of follow-up. The auricular injury classification [6] was used to classify the extent of the injury, assessed on the photo obtained during primary survey (Table 1). This photo was used to identify the involved subsides of injured auricle.

We have reported the clinician’s experience in terms of years in otorhinolaryngology residency, supplemented with the final category qualified otorhinolaryngologist.

### Success classification

**Fig. 1:**
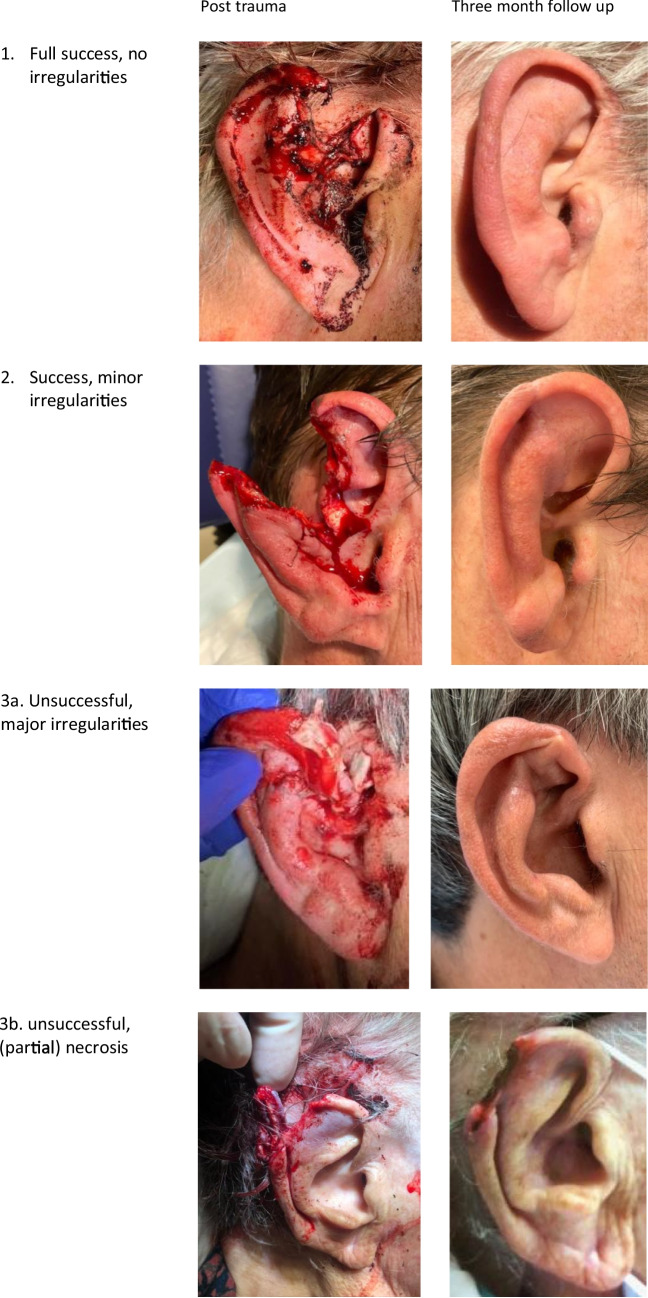
This figure we introduce our revised classification system to assess postoperative success following primary closure of an auricular laceration through some examples. Four classes were developed: (1) full success, no minor irregularities, (2) partial success, only minor irregularities, (3a) unsuccessful, major irregularities or (3b) unsuccessful, (partial) necrosis

To evaluate the achieved results after 3 months a classification system with four classes was developed: (1) full success, no minor irregularities, (2) partial success, only minor irregularities, (3a) unsuccessful, major irregularities or (3b) unsuccessful, (partial) necrosis (Fig. 1). This classifications system is based on the classification used by Steffen et al. [12], extended with the classification of (1) full success, no minor regularities.

Three independent otolaryngologists assessed the success based on the photo obtained three months after the primary survey. In the event of a difference in success classification, agreement was obtained after mutual consultation.

### Statistical analysis

Data are expressed as numbers. IBM SPSS Statistics 29 was used for the analysis. Correlation between patient- and injury characteristics and treatment success three months after primary survey was measured using Pearson Chi-square test (between categorical data) and ANOVA-one way test (between categorical and nominal data). Effect size was measured using a bivariate Pearson model and paired sample T-tests. Interobserver variability was measured using interobserver percent agreement and Fleiss’ kappa coefficient [12, 13].

## Results

A total of 39 patients were presented with an auricle laceration in our hospital during the study period. Of these 39 patients, 12 (31%) were female. The mean age of all patients was 45 years (range 4–98 years). A left sided laceration was presented in 21 patients (54%). None of our patients had a double-sided ear laceration.

**Fig. 2:**
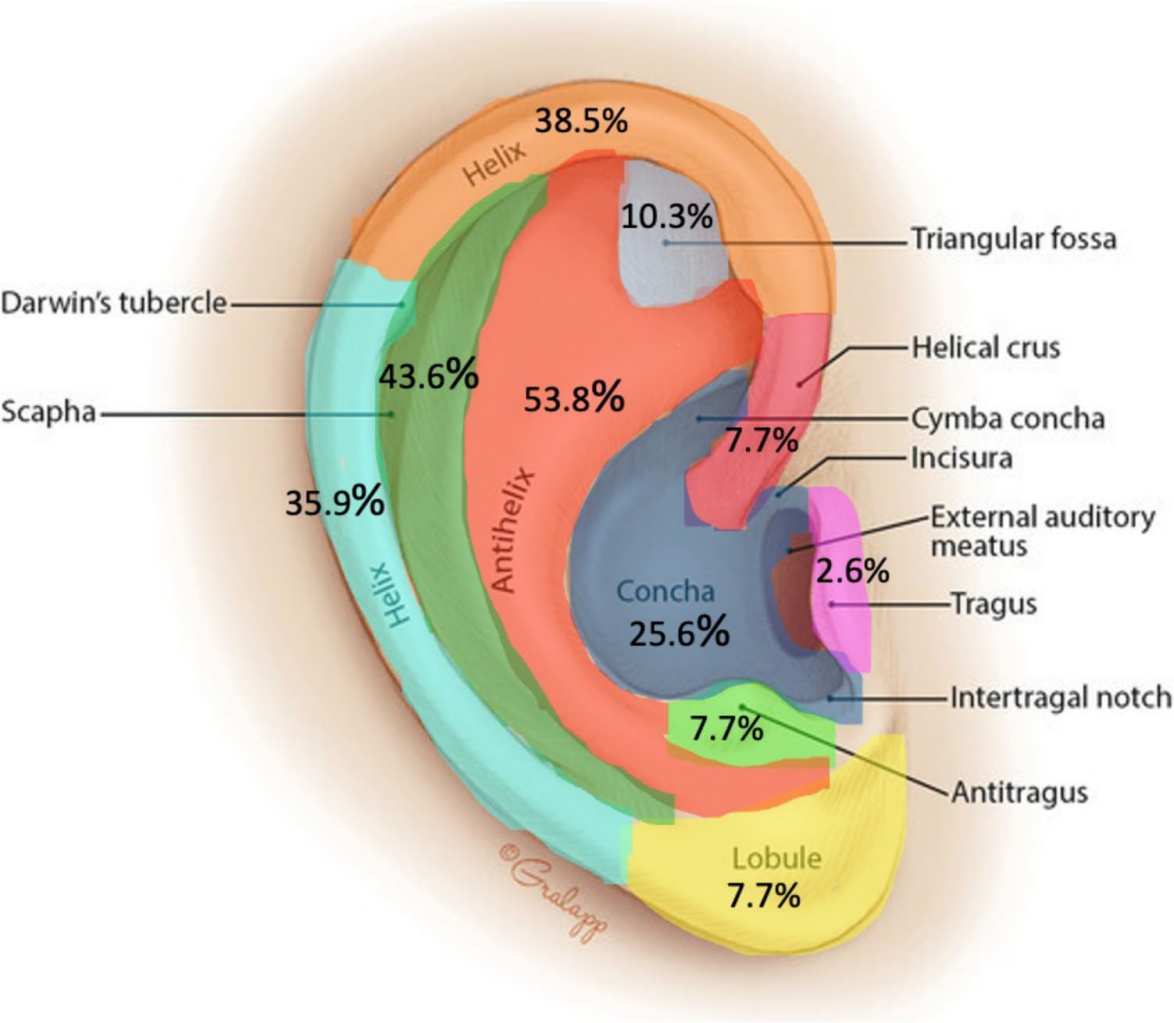
This figure displays the percentages of the anatomical subsites of the auricle involved on primary survey. Multiple subsites can be involved in a single laceration

An overview of the subsites of the auricle involved at primary survey is displayed in Fig. 2. Usually more than one subsite is involved in our series. The helix was involved in 24 of our patients (62%). We have subdivided the helix in an inferior helix (inferior of Darwins tubercle) (14/39 (39%)), superior helix (15/39 (38.5%)) and the helical crus (3/39 (7%)) as displayed in Fig. 2. The scapha (17/39, (43.6%)) and the antihelix (21/39 (53.8%)) were also frequently involved in auricular trauma.

Table 1 displays baseline characteristics of the subjects. Based on the Auricle Injury Classification by Lavasani et al. 12/39 patients had a cartilage sparing injury, 24/39 patient had a partial avulsion with wide pedicle and 3 of the 39 patients had a partial avulsion with narrow pedicle [1]. None of our patients had a complete avulsion.

After 3 months of follow up 28 of the 39 patients (71.6%) showed up for a visit in the outpatient clinic to obtain the 3 month follow up picture. Of the 11 patients lost to follow up, 2 patients died due to other disease, 1 patient moved abroad, 4 patients were tourists and had post-surgical controls in their country of origin and 4 patients did not respond on repeated requests for their post-surgical appointment.

Seventeen out of 28 patients (60.7%) were scored “full success without irregularities” (1), 9 (32.1%) scored partial “success with minor regularities” (2), 1 (3.6%) scored “unsuccessful with major irregularities” (3a) and 1 (3.6%) scored “unsuccessful with partial necrosis” (3b).

Baseline characteristics, risk factors, cause of the ear trauma and the auricular injury classification were not correlated (*p* > 0.05) to the 3 month follow up success classification. Helical involvement was correlated to the 3-month success classification (*p* = 0.005, r = 0.519). The positive effect size implies that the helical involvement negatively predicts the surgical result. If we subdivide the helix in the inferior helix (p = 0.028, r = 0.424), the superior helix (*p* = 0.048, r = −0.049) and helical crus (*p* = 0.034, r = 0.427) we had significant correlation but weak effect size. The time between trauma and surgery, experience of the physician, use of coagulation for hemostasis, suture material, package material used were all not significantly correlated to the surgical outcome at 3 months follow up.

The interobserver percent agreement was 88%. The Fleiss’ kappa coefficient was 0.570, *p* < 0.001.

## Discussion

To the best of our knowledge, we are the first to study the surgical results of primary closure of auricle lacerations with a reproducible success classification system assessed 3 months after primary closure. We demonstrate that most patients referred to the emergency department with an auricular laceration have excellent post-surgical results at 3 month follow up (success classification 1 and 2 (92.8%)). This is in accordance with the results presented by Spira (1963) and Bardsley (1983) [10, 11]. We found no correlation between the physicians’ experience and the surgical outcome.

In this study, we introduce a revised classification system to assess postoperative success following primary closure of an auricular laceration. This classification system is based on the classification system introduced by Steffen et al. [14]. We have added the category"full success, no irregularities"because in our study, patients with a complete avulsion of the auricle are absent. In our opinion, it would be more accurate to add this category to a revised classification system to evaluate the success rate of primary closure of ear lacerations. The addition of a more critical category is based on the high success rates reported in previous studies [10]

The revised classification system is a subjective assessment of the success of the primary closure of an auricular laceration. We have found a percent agreement between three independent observers of 88%. The Fleiss’ Kappa coefficient can compare interrater reliability across more than two observers [13]. We determined a Fleiss’ Kappa coefficient of 0.57, representing moderate agreement.

The revised success classification system introduced in this article will help clinicians to assess their success rates and may help future researchers to discover potential confounders for unsuccessful ear laceration repair.

Unlike the findings of Steffen et al. in 2007, the most common incident of an auricle laceration in our study group was a home accident [7]. Compared to the case series of Steffen et al. our patient population had a higher mean age and a higher percentage of elderly. The increased presence of elderly may serve as an explanation of higher incidence of home accidents as traumatic mechanism [15].

Involvement of the helix was associated with inferior surgical success at three month follow up. We have tried to subdivide the helix to investigate potentially more vulnerable subsites of the helix associated with inferior post-surgical results after auricular lacerations. We could not find a helical subside with a significant effect size. Tension of skin and underlying cartilage in the auricular region is orientated perpendicular to the long axis at the base of the auricle and parallel at the apex [16]. This pattern is likely to play a role in the clinical outcome after reconstruction of auricle lacerations with helical involvement.

Cadaveric studies have shown that the vascular pattern of the auricle is provided by a complex network originated from the posterior auricular artery (PAA) and the superficial temporal artery (STA) [4, 5]. Branches of the PAA and STA collateralize through perforations in the cartilage and cover the edge of the helical rim, eventually creating a network of random skin collaterals [4, 5]. We think this two-sided blood supply from branches of the PAA and the STA is responsible for the high success rate of primary closure of auricle lacerations. The laceration classification introduced by Lavasani et al. is based on the blood supply [1]. In our study population this trauma classification system was not correlated (*p* = 0.122) to the revised success classification system introduced in this study. This might be due to the small sample size. There is an overlap between the helical involvement correlated in our study population and a narrow pedicle as a suggested negative confounder of surgical success after auricle laceration by Lavasani et al. For this reason, we think the auricle laceration classification of Lavasani et al. should be studied in a future study as a potential negative confounder of ear laceration repair.

The need for antibiotic prophylaxis in head and neck injuries is under debate [6]. Since most wounds are contaminated and the possible irreversible effects of a perichondritis to the shape of the auricle, we administered one week of amoxicillin-clavic acid to cover for *Pseudomonas aeruginosa and Staphylococcus aureus* infections in all patients [6]. Therefore, our study does not provide an answer about the added value of antibiotic prophylaxis in patients with auricular lacerations.

Two of our included patients had an unsuccessful surgical result. The first patient was 98-year-old female with dementia and a laceration of the upper- and inferior helical ridge with almost no remaining pedicle. Most likely the near total avulsion of the helical ridge, causing almost no residual blood supply, had a negative effect on the wound healing. Another unsuccessful surgical outcome involved a 50-year-old male patient who sustained a tear extending from the helical root to the conchal cartilage. During the three-month follow-up, a noticeable step in the cartilage and a discolored scar were observed. It is likely that one of the sutures at the helical root did not properly secure the cartilage.

Our study is limited by the small sample size. In the six years duration of this single center study only 39 patients were referred to our department. To find predictors for successful surgical outcome after primary closure of an auricular laceration a multicenter study is needed. Our introduced revised classification system can be used to evaluate the success rate in such a future study.

## Conclusion

Primary closure of ear lacerations results in a high success rate. We have introduced a revised classification system to qualify the success of treatment of the auricle laceration. Involvement of the helix is correlated with inferior surgical results three months after surgical closure.

## Data Availability

The data that support the findings of this study are available from the corresponding author upon reasonable request.

## Abbreviations

PTA: Posterior auricular artery
STA: Superficial temporal artery
